# Pharmaceutical preparation of *Saubhagya Shunthi Churna*: A herbal remedy for puerperal women

**DOI:** 10.4103/0974-7788.59940

**Published:** 2010

**Authors:** Khushbu Shukla, Manjari Dwivedi, Neeraj Kumar

**Affiliations:** *Department of Rasa Shastra, Institute Medical Sciences Banaras Hindu University, Varanasi - 221 005, India*; 1 *Department of Prasuti Tantra, Institute Medical Sciences Banaras Hindu University, Varanasi - 221 005, India*

**Keywords:** Ayurveda, *Churna* (powder), *saubhagya shunthi paka*, *paka* (semi-solid), puerperium

## Abstract

**Background:**

In the last few decades, there has been exponential growth in the field of herbal remedies. Pharmacopoeial preparations like *avleha or paka* (semi-solid), *swarasa* (expressed juice), *kalka* (mass), *him* (cold infusion) and *phanta* (hot infusion), *kwatha* (decoction) and *churna* (powder) form the backbone of Ayurvedic formulations. Newer guidelines for standardization, manufacture, and quality control, and scientifically rigorous research will be necessary for traditional treatments. This traditional knowledge can serve as powerful search engine that will greatly facilitate drug discovery.

**Purpose:**

The aim of the present study is to standardize *Saubhagya Shunthi Paka* in *churna* (powder) form. The powder form makes this traditional drug more stable for long-term storage and hence, easier to preserve.

**Materials and Methods:**

*Saubhagya Shunthi Paka* is an ayurvedic formulation containing *Shunthi* (*Zingiber officinalis*) as one of its chief ingredients. The basic preparation of this drug is a semisolid. We checked the microbial load and nutrient values (using International Standard IS and Association of Official Analytical chemists AOAC methods)

**Results:**

The powdered form of *Saubhagya Shunthi Churna* yielded a weight loss of approximately 17.64% of the total weight of ingredients. The total energy of *Churna* (calculated based on nutrient content) was found higher over *Paka*.

**Conclusion:**

*Saubhagya Shunthi Churna* may be a good therapeutic and dietary medicine for Indian women, which may be easily prepared at home.

## INTRODUCTION

Ayurveda, the herbal-based system of medicine is now well recognized not only in India, but also in the Western world. With the growing need for safer drugs, attention has been drawn to the quality, efficacy, and standards of Ayurvedic formulations.[1] In India, Ayurveda involves the eight principal branches of medicine: Pediatrics, gynecology, obstetrics, ophthalmology, geriatrics, otolaryngology, general medicine, and surgery. During the past few decades, there has been a growing recognition of reproductive health issues particularly, in women. Every year, at the global level, some eight million women suffer pregnancy-related complications and over half a million die, 99% of them in the developing countries.[2] Problems that are specific to women's reproductive process can be divided into two. Firstly, problems occurring during pregnancy, delivery, and the puerperium, referred to in the medical literature as obstetric (maternal) morbidity. Secondly, problems occurring with nonpregnant women and outside the puerperal period of six weeks, known as gynecological morbidity.[3] Women's health is a basic need for society as it affects the progeny. A woman who has just given birth to a baby along with the placenta is called “*Sutika*” in Ayurveda. During puerperium, the woman faces many problems like fever, diarrhea, edema, colic pain, abdominal distension, loss of strength, drowsiness, anorexia, delirium, and other diseases that are caused by the vitiation of *kapha* as well as *vata* which appear during puerperium. The classical concept of Ayurveda defines the ways to maintain ‘*Vata*’, ‘*Pitta*,’ and ‘*Kapha*’ in a balanced state to prevent diseases.[4] These are difficult to cure because of the decrease in muscle tissue and strength in women during the puerperal period. Diseases associated with the puerperal period are called *Sutika Roga* (puerperal diseases).[5]

Ayurveda mentions specific drugs that are given for a definite duration along with specific dietetic regimens for puerperal women. *Saubhagya Shunthi Paka* is an Ayurvedic herbal formulation containing *Shunthi* as the chief ingredient. It alleviates anxiety, stress and is a natural pain reliever known to contain about 17 crude drugs.[6]

### Soubhagya shunthi churna: An overview

Ayurveda uses various formulations such as solid dosage forms (pills, powders), liquid dosage forms (*asavas*, *aristhas*), and semisolid dosage forms (*ghritas*, *avlehas*, *and paka*). Pharmacopoeial preparations like *swarasa* (expressed juice), *kalka* (mass), *him* (cold infusion) and *phanta* (hot infusion), *kwatha* (decoction), and *Churna* (powder) form the backbone of Ayurvedic formulations.[7] *Paka* is a semisolid preparation of drugs prepared by the addition of jaggery or sugar.[4]

*Saubhagya Shunthi Paka* is a classical preparation from the Ayurvedic text, *“Yoga Ratnakar*.*”* It is a very useful drug for puerperal women because it contains all the nutrients which are required during this period and can be easily prepared at the home. The combination of *Saubhagya Shunthi Paka* with *Dashamoolarishta* has a potent effect on postpartum women by helping to fulfil their body requirements and to restore their bodies to normalcy. It is known to improve digestion and relieves debility following delivery. It works well as a postnatal tonic and facilitates normal involution of the uterus, besides enhancing the production of milk.

*Saubhagya Shunthi Paka*, is appropriate to review is not very well known it, but because of its usefullness this traditional drug. As the *paka* preparation cannot be stored for long periods, we have formulated it in the *Churna* form, which retains the same qualities but can be preserved for longer periods. Thus, the formulation can be manufactured in large scale to be marketed as an Ayurvedic medicine.

*Saubhagya Shunthi Paka* consists of 17 herbal ingredients including, which have their individual health promotive effects; and their roles in puerperium have been discussed below:

- Goghrita (cow's ghee)
- Khoya (concentrated milk)
- Sita (jaggery) (*Saccharum officinarum*)
- Shunthi (*Zingiber officinale*)
- Mishriya (*Foeniculum vulgare*)
- Mustaka (*Cyperus rotundus*)
- Javitri (*Myristica fragrans*)
- Krishna-jeeraka (*Bunium persicum*)
- Sweta-jeeraka (*Cuminum cyminum*)
- Nagkeshar (*Mesua ferra*)
- Marica (*Piper nigrum*)
- Dhanyaka (*Coriandrum sativum Linn.*)
- Pippali (*Piper longum*)
- Indrjaua (*Holarrhena antidysenterica*)
- Vidang (*Embelia ribes*)
- Tejpatra (*Cinnamomum tamala*)
- Ela (*Elattaria cardamom*)

The objective of the present study was to develop a more stable churna formulation by using the same traditional medicinal herbs.

## MATERIALS AND METHODS

### Estimation of moisture content routine procedure

The moisture content of the raw materials used in preparation of the *Soubhagya Shunthi* was estimated as follows:

1. Weights of raw material samples and weights of Petri-plates were taken separately.
2. The fresh samples were taken in the Petri-plates.
3. The Petri-plates were incubated in the oven for 24 hours at 105°C.
4. The samples were removed from the oven and cooled to room temperature.
5. Again the weights of the raw material along with the Petri-plates were measured.

Moisture content was calculated by using the formula

$$(\mathrm{Weight}\;\mathrm{of}\;\mathrm{Petri}-\mathrm{plates}+\mathrm{Weight}\;\mathrm{of}\;\mathrm{raw}\;\mathrm{material})-\frac{\mathrm{Weight}\;\mathrm{of}\;\mathrm{oven}-\mathrm{dried}\;\mathrm{sample}}{\mathrm{Weight}\;\mathrm{of}\;\mathrm{oven}-\mathrm{dried}\;\mathrm{sample}}\;\mathrm{\times 100}$$

### Preparation of *Saubhagya shunthi churna*

All the raw materials required for the preparation were weighed in grams [Table 1] and powdered separately in a pulverizer and then weighed again.

1. *Khoya* was taken in a vessel and heated with “*Madhyanagni*” (medium intensity fire) with the addition of a little *Goghrita* until it became brown in color.
2. *Goghrita* was taken in another vessel and mixed with the powder of *Shunthi* before frying the preparation properly.
3. All the *Prakshepya Dravya* drugs were taken in their powdered forms, *i.e., Khand (jaggery), Mishreya (Foeniculum vulgar), Dhanyaka (Coriandrum sativum), Vidanga (Embelia ribes), Maricha (Piper nigrum), Swetajeeraka (Cuminem cyminum), Krishnajeeraka (Nigella sativa), Javitri (Myristica fragrans), Pippali (Piper longum), Ela (Elattaria cardamom), Tejpatra (Cinnamomum tamala), Nagkeshar (Mesua ferra), Indrajau (Holarrhena antidysenterica), Musta (Cypurus rotundus)* along with the fried *Khoya* (condensed milk) and fried “*Shunthi* preparation”.
4. All the contents were properly mixed to obtain *Saubhagya Shunthi Churna*.

**Table 1:** Ingredients of *Saubhagya shunthi churana*

Materials	Weight (g)
Cow's *ghee*	1000
*Khoya*	1000
*Khand*	2500
*Shunthi*	450
*Mishreya*	250
*Dhanyaka*	150
*Vidanga*	50
*Maricha*	50
*Swetajeeraka*	50
*krishnajeeraka*	50
*Javitri*	50
*Pippali*	50
*Ela*	50
*Tejpatra*	50
*Nagkeshar*	50
*Indrajau*	50
*Musta*	50

### Assessment of nutritive value of churna and paka preparations of *Saubhagya shunthi*

The samples of both the forms (*Churna* and *Paka*) of the drug were sent to the ‘Regional food and Research Analysis Centre, Lucknow’, where certain tests were performed to investigate their nutritional value. They used the ‘IS method’ and ‘AOAC Method’ as follows:

Calculation of Total Energy = (Estimated value of Protein × 4) + (Estimated value of Fat × 9) + (Estimated value of Carbohydrate × 4)

### Shelf-life analysis of *Saubhagya shunthi churna and paka*

This test was performed to check the microbial load of both the samples in our own laboratory. The samples were incubated in Yeast Extract Mannitol (YEM) medium for 36 hours along with plain YEM medium as a control.

## RESULTS

The color of *Shunthi* was yellowish at the start and during the process and became brown after completion of the process. The weight loss of the ingredients after pulverizing into the powder form was 12.7% [Table 2]. *Shunthi* absorbed almost the entire amount of *Goghrita* at the start.

**Table 2:** Weight loss of ingredients during grinding of herbs

Name of ingredients	Initial weight (g)	Final weight (g)	Loss of weight (g)
*Shunthi*	450	425	25
*Mishreya*	250	220	30
*Dhanyaka*	150	110	40
*Vidanga*	50	45	5
*Maricha*	50	45	5
*Swetajeeraka*	50	40	10
*Krishnajeeraka*	50	45	5
*Javitri*	50	40	10
*Pippali*	50	45	5
*Ela*	50	47	3
*Tejpatra*	50	35	15
*Nagkeshar*	50	40	10
*Indrajau*	50	35	15
*Musta*	50	40	10
*Total powdered herbs*	1400	1212	188

When all the contents were mixed with the fried condensed milk and the *Shunthi* fried with *Goghrita*, the final preparation of the drug was observed to be brown in color.

The total weight loss of the drug during the final preparation was 17.64% (this means that 4.94% of the weight loss was recorded during the formulation of the drug) [Table 3]. Moisture content of *Pippali* was found to be the highest (3.55) whereas it was the lowest in *Krishna jeeraka* (1.49). The moisture content of two ingredients showed negative values: –6.00 and –2.85 for *Vidang* and *Tejpatra* respectively [Table 4].

**Table 3:** Total loss of weight of ingredients during preparation of drug

Name of ingredients	Initial weight (g)	Final weight (g)	Loss of weight (g)
Powdered herbs	1400	1212	188
Condensed milk	1500	1000	500
Cow's butter	1000	1000	0
Total	3900	3212	688

**Table 4:** Total loss of moisture content of ingredients

Sample	Wt. of FS^1^ (g)	Wt. of PP^2^ (g)	Wt. of ODS^3^ (g)	Moisture content (%)
*Shunthi*	29.44	47.67	75.2	2.539894
*Marica*	29.89	29.56	57.56	3.28353
*Dhaniya*	12.63	41.52	52.85	2.459792
*Indrajau*	12.36	38.73	50.29	1.590774
*Jeera*	10.64	33.64	43.61	1.536345
*Krishna jeeraka*	12.01	50.59	61.68	1.491569
*Soufa*	14.16	36.56	49.35	2.776089
*Motha*	18.09	41.81	58.51	2.375662
*Ila*	19.28	36.6	47.74	17.05069
*Nagkesher*	11.22	38.23	48.56	1.832784
*Pippali*	15.85	40.48	54.4	3.547794
*Vidang*	10.01	44.16	57.63	–6.00382
*Tejpatra*	4.49	15.96	21.05	–2.85036
*Javatri*	5.72	15.96	21.19	2.312412

Weight of fresh sample (raw material used in drug preparation)-Wt. FS; Weight of Petri-plates-Wt. PP; Weight of oven dry sample-Wt. ODS

The total energy of *Churna* (489.0 Kcal/100 g) was higher than that of *Paka* (426.0 Kcal/100 g) because the carbohydrate value of *Churna* is 41 g more than that of *Paka*. Calcium content was approximately the same for both preparations whereas iron and protein were higher in *Churna* in comparison with *Paka* [Table 5].

**Table 5:** Estimation of nutritive value of *Saubhaguya Shunthi Paka* and C*hurna*: A comparative analysis

Nutrients	*Saubhagya Shunthi Paka* (delivery/per gram)	*Saubhagya Shunthi Churna* (delivery/per gram)
Iron	10.5 mg	17.04 mg
Protein	6.8 g	7.15 g
Fat	25.7 mg	14.11 mg
Carbohydrate	42.5 g	83.5 g
Calcium	212.02 mg	211.39 mg
Vitamin B^12^	0.5 mcg	0.5 mcg/100 g
Total Energy	426.0 Kcal	489.6 Kcal/100 g

**Figure 1:**
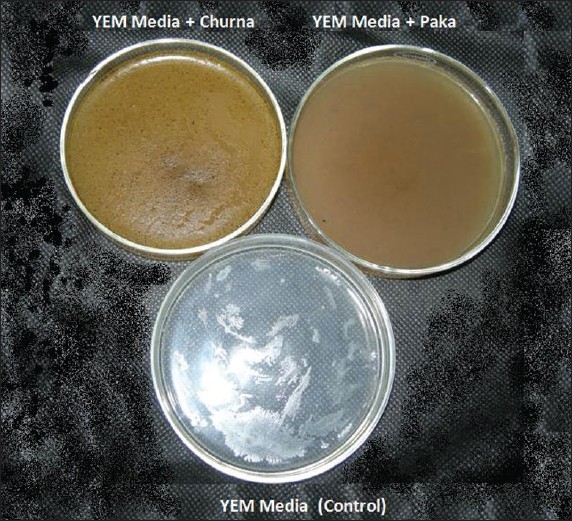
shelf-life study of *Saubhagya shunthi churna* and *paka* after 36 hour incubation

In the shelf-life, we found no contamination in either of the samples. (*Churna* preparation was two years old, the *Paka* was only four months old) [Figure 1].

## DISCUSSION

Ayurveda is practised widely in India, Sri Lanka, and other countries, and has a sound philosophical and experiential basis.[26,27] Atharvaveda (around 1200 BC), Charak Samhita, and Sushrut Samhita[28] (1000–500 BC) are the main classics that give a detailed description of over 700 herbs. Today the Government of India has formed stringent to regulate issues related to quality, safety, efficacy, and practice of herbal medicine.[29] With a unique holistic approach, Ayurvedic medicines are usually customized to the individual's constitution.[30]

Standardization and development of reliable quality protocols for Ayurvedic formulations using modern techniques of analysis is extremely important.[31] Standardization should be done by using appropriate amounts of raw materials, followed by in-process control and shelf-life analysis with authentic clinical trials.[32]

*Shunthi* is used in folk medicine for relief from many ailments, especially nausea, motion sickness, and other gastrointestinal disorders.[33] *Churna* and *paka kalpana* both have similar effect in *sutika kala*. However the present study was focused on *churna kalpana* because of the short shelf- life of *paka kalpana*. According to the ‘Ayurvedic Formulary of India’, the *Paka* (*Avaleha*) should be used within one year only,[34] whereas *Churna* is safe for use even after two years. The nutritive value is also an important reason for the preference of *Churna* to *Paka*. In light of this information, *Saubhagya Shunthi Churna* to be used for this study was prepared from the same ingredients as those described in *Yoga Ratnakar*.

## CONCLUSION

*Saubhagya Shunthi* can be prepared in both forms, *i*.*e*., *churna* (Powder) and *paka* (semisolid). Although both preparations show the same effect in *Sutika Kala*, the *churna* can be seen to be better than the *paka* form due to its longer shelf-life and comparatively higher total energy. *Saubhagya Shunthi Churna* may be a good therapeutic and dietary medicine for Indian women, which may be prepared at home easily. This traditional formulation can provide novel insights into the drug discovery and development process., This drug can be useful for the pharmaceutical companies searching for economically valuable natural products.

The design of a new drug necessitates the study of the effects of a drug. Thus, the clinical benefits of this ayurvedic drug over standard therapy should be extremely convincing. Hence, there is a need for further study to evaluate the effects of the drug by a case control study and to elucidate its complete mechanism of action.
